# Post-marketing safety concerns with lumateperone: a pharmacovigilance analysis based on the FDA adverse event reporting system (FAERS) database

**DOI:** 10.3389/fphar.2024.1389814

**Published:** 2024-05-09

**Authors:** Dan Zhao, Wangxin Zhang, Yan Liu, Zhaojun Yan

**Affiliations:** ^1^ Department of Psychosomatic Medicine, Affiliated Hospital of Shandong University of Traditional Chinese Medicine, Jinan, China; ^2^ Shandong First Medical University and Shandong Academy of Medical Sciences, Tai’an, China

**Keywords:** adverse event, FAERS database, lumateperone, disproportionality, pharmacovigilance

## Abstract

**Objective:**

Lumateperone, a novel antipsychotic drug that was granted by the Food and Drug Administration (FDA) approval in December 2019, remains insufficiently explored for its adverse event profile. This study used the FDA Adverse Event Reporting System (FAERS) database to explore its potential safety issues.

**Methods:**

This study conducted a retrospective analysis of FAERS data from the fourth quarter of 2019 to the third quarter of 2023, extracting reports related to lumateperone. Disproportionality analysis using Reporting Odds Ratio (ROR) and Bayesian Confidence Propagation Neural Network (BCPNN) algorithms was employed to detect signals of adverse events (AEs).

**Results:**

Our research processed 4,777 pertinent AE disclosures related to lumateperone, unveiling 125 signals that satisfied both ROR and BCPNN evaluative benchmarks across 26 System Organ Classes (SOCs). Intriguingly, 108 of these signals were categorized as unanticipated, spotlighting notable psychiatric manifestations such as mania (ROR = 73.82, 95% CI = 57.09–95.46; IC = 6.16, IC025 = 4.49), and hypomania (ROR = 34.74, 95% CI = 15.54–77.64; IC = 5.10, IC025 = 3.43), alongside non-psychiatric phenomena like urinary retention (ROR = 3.59, 95% CI = 1.80–7.19; IC = 1.84, IC025 = 0.18) and serotonin syndrome (ROR = 8.69, 95% CI = 4.81–15.72; IC = 3.11, IC025 = 1.45).

**Conclusion:**

This research provides real-world safety data on lumateperone post-marketing and is an important supplement to the information from clinical trial studies. Healthcare professionals should be vigilant for the risk of a manic switch in patients with bipolar depression who are administered lumateperone. More epidemiological studies are needed in the future to explore and further evaluate the risk-benefit issue of lumateperone.

## 1 Introduction

In December 2019, the Food and Drug Administration (FDA) authorized the market introduction of lumateperone, a novel therapeutic agent heralding a significant advance in the treatment of psychiatric disorders, notably schizophrenia and the depressive episodes associated with bipolar I and II disorders. Lumateperone is a novel atypical antipsychotic drug that exerts its antipsychotic and antidepressant effects by modulating the pathways of serotonergic, dopaminergic, and glutamatergic neurotransmission (Kumar et al., 2018; Blair, 2020; Abuelazm et al., 2023). This pharmacological duality offers a comprehensive therapeutic avenue for individuals grappling with multifaceted psychiatric disorders and provides important insights into the pathomechanisms of schizophrenia and bipolar disorder. The drug’s demonstrated potential to ameliorate the positive, negative, and cognitive manifestations of schizophrenia, along with its effectiveness in controlling depressive episodes in bipolar disorder, further accentuates its transformative impact on therapeutic psychiatry (Edinoff et al., 2020; Jawwad et al., 2022; Abuelazm et al., 2023; McIntyre et al., 2023). Furthermore, clinical studies demonstrated that lumateperone, due to its low D2 receptor occupancy, significantly reduces the probability of experiencing extrapyramidal symptoms (EPS), which are frequent adverse effects of other antipsychotic drugs (Durgam et al., 2020).

Despite its unique pharmacological effects and promising applications, lumateperone still poses potential safety risks in real-world applications. The main adverse events reported in current epidemiological studies and pre-marketing clinical trials include somnolence, nausea, sedation, fatigue, and constipation (Calabrese et al., 2021; Maini et al., 2021). However, it is important to note that these studies may not fully reflect the drug’s safety profile due to limited sample sizes, short observation periods, and different inclusion criteria for participants (Sakaeda et al., 2013; Ford and Norrie, 2016).

Consequently, there is a pressing need for pharmacovigilance studies that leverage real-world data to comprehensively assess the safety of lumateperone. Such studies are instrumental in identifying, quantifying, and understanding the adverse effects of new medications, ensuring the wellbeing of the patient population. In this context, our research used data from the FDA Adverse Event Reporting System (FAERS), which holds significant implications for the pharmacovigilance of lumateperone. The FAERS database, a rich repository of post-marketing safety reports, provides an invaluable resource for monitoring the adverse events of medications in an authentic world setting. With the analysis of these data, our study aims to elucidate the safety profile of lumateperone and contribute to the body of evidence on its adverse events. This endeavor not only enhances our understanding of lumateperone’s safety but also underscores the importance of ongoing post-marketing drug safety surveillance. Through this pharmacovigilance research, we aspire to offer useful information for clinical practice, guide therapeutic decisions, and ultimately safeguard patient health.

## 2 Materials and methods

### 2.1 Data source and data processing

Our research data was sourced from the FAERS database, one of the significant pharmacovigilance databases, encompassing all reports collected by the FDA from 2004 to the present. These reports include not only adverse drug reactions and medication error information but also product quality complaints leading to adverse drug events. Reporting to the FAERS database via the FDA website is unrestricted by time or geography and is open to healthcare professionals, consumers, and product manufacturers alike. The FAERS database serves as a surveillance system for the quality and safety of FDA-approved drugs, offering evidence for clinical safety in medication use. It represents a compelling data source of pharmacovigilance data for early detection and the prompt description of unanticipated toxicity (Raschi et al., 2019; Chrétien et al., 2021). All adverse event medical terms are coded, categorized, and standardized according to the Medical Dictionary for Regulatory Activities (MedDRA), identifying specific signs and symptoms of clinical entities based on preferred terms (PTs). The FAERS database is publicly released quarterly and is distributed across seven datasets: demographic information, use/diagnosis indications, reporting sources, adverse event records, treatment outcomes, drug usage records, and the start and end dates of drug therapy. The database can be accessed by the public through this web address: https://fis.fda.gov/extensions/FPD-QDE-FAERS/FPD-QDE-FAERS.html. Since lumateperone was approved by the FDA in December 2019, all reports listing this medication as the primary suspected (PS) drug from the fourth quarter of 2019 to the third quarter of 2023 were included in our analysis, without restrictions on gender, age, or nationality. Due to potential issues of duplicate reporting and non-standard reports in the submission process, the downloaded raw data requires cleaning to ensure the reliability and quality of the data. For instance, when two reports share the same CASEID (case ID), we retain the report with the most recent FDA_DT (report date). If two reports have identical CASEID and FDA_DT, the report with the higher PRIMARYID (report ID) is chosen.

### 2.2 Study design and statistical analysis

As shown in Supplementary Table S1, this investigation adopts a case-control study framework to elucidate the relationship between pharmaceutical interventions and the incidence of adverse events (AEs) (Chavant et al., 2011). This design is particularly suited for examining rare events and for establishing a temporal connection between drug exposure and subsequent AEs. Cases were reports of adverse events recorded for “LUMATEPERONE,” “LUMATEPERONE TOSYLATE,” or “CAPLYTA,” whereas controls were reports of adverse events for all other medications in FAERS (Sakaeda et al., 2013). To augment the reliability and robustness of our findings, we used the Reporting Odds Ratio (ROR) and Bayesian Confidence Propagation Neural Network (BCPNN) techniques to assess the association between pharmaceuticals and adverse events (Caster et al., 2020; Gastaldon et al., 2021). Detailed formulations and threshold parameters are delineated in Supplementary Table S2. A signal is generated when the criteria for both methodologies are concurrently met, indicating a significant association. The ROR is calculated as a measure of disproportionality by the odds of reporting a particular adverse event for a drug versus the odds of reporting that event for all other drugs (Noguchi et al., 2021). For the BCPNN, the information component (IC) is computed, which quantifies the extent of linkage between drugs and adverse events using Bayesian inferential statistics (Bate et al., 1998). Both methods operate on the principle that a larger value indicates a stronger signal and a greater likelihood of a true relationship between the drug and the adverse event (Zou et al., 2023). The significance of the ROR is established by evaluating the 95% confidence intervals (CIs), where significance is ascertained if the ROR’s lower confidence limit exceeds 1 and there are at least three reports of associated adverse events (Zou et al., 2023; Jiang et al., 2024). Concerning BCPNN, a value of IC025 exceeding zero is indicative of a meaningful association.

The study also conducted subgroup analyses to mitigate the impact of demographic characteristics on the outcomes. Due to the low number of cases in the <18 and 65–85 age subgroups, age was not analyzed as a subgroup. Within the gender subgroups, ROR (95% CI) and IC (IC025) were calculated separately. A disproportionality signal was considered when both detection methods met their threshold criteria. Additionally, we selected risperidone, a second-generation antipsychotic medication with similar indications to lumateperone, for a comparative disproportionality analysis on a set of significant adverse drug events. This approach improves the specificity of detecting adverse event signals related to lumateperone, reducing the influence of potential confounding factors.

In our analysis, we carefully handle the dilemma of incomplete or inaccurate date entries in the FDA Adverse Event Reporting System (FAERS) database to determine the time to onset of an adverse event attributed to lumateperone. We first excluded records that lacked precise date details or provided only the year of occurrence. For entries with only partial dates (years and months), we supplemented them with the midpoint of the corresponding month, thus ensuring uniform criteria for calculating the time interval between the initiation of treatment with lumateperone and the occurrence of an adverse event report. This time interval was measured in days and derived from the difference between the date of treatment initiation (START_DT in the THER file) and the date of the adverse event (EVENT_DT in the DEMO file). Moreover, we performed the Weibull Proportionality Test, a statistical method widely used for modeling time-to-event data, on the changes in the incidence of adverse events after administration of lumateperone. The analysis used the Weibull shape parameter (WSP) to explore the hypothesis that the incidence of adverse events changes over time (Kinoshita et al., 2020). The median and interquartile range for adverse event induction times were calculated to determine the central tendency and variability of the distribution.

**FIGURE 1:**
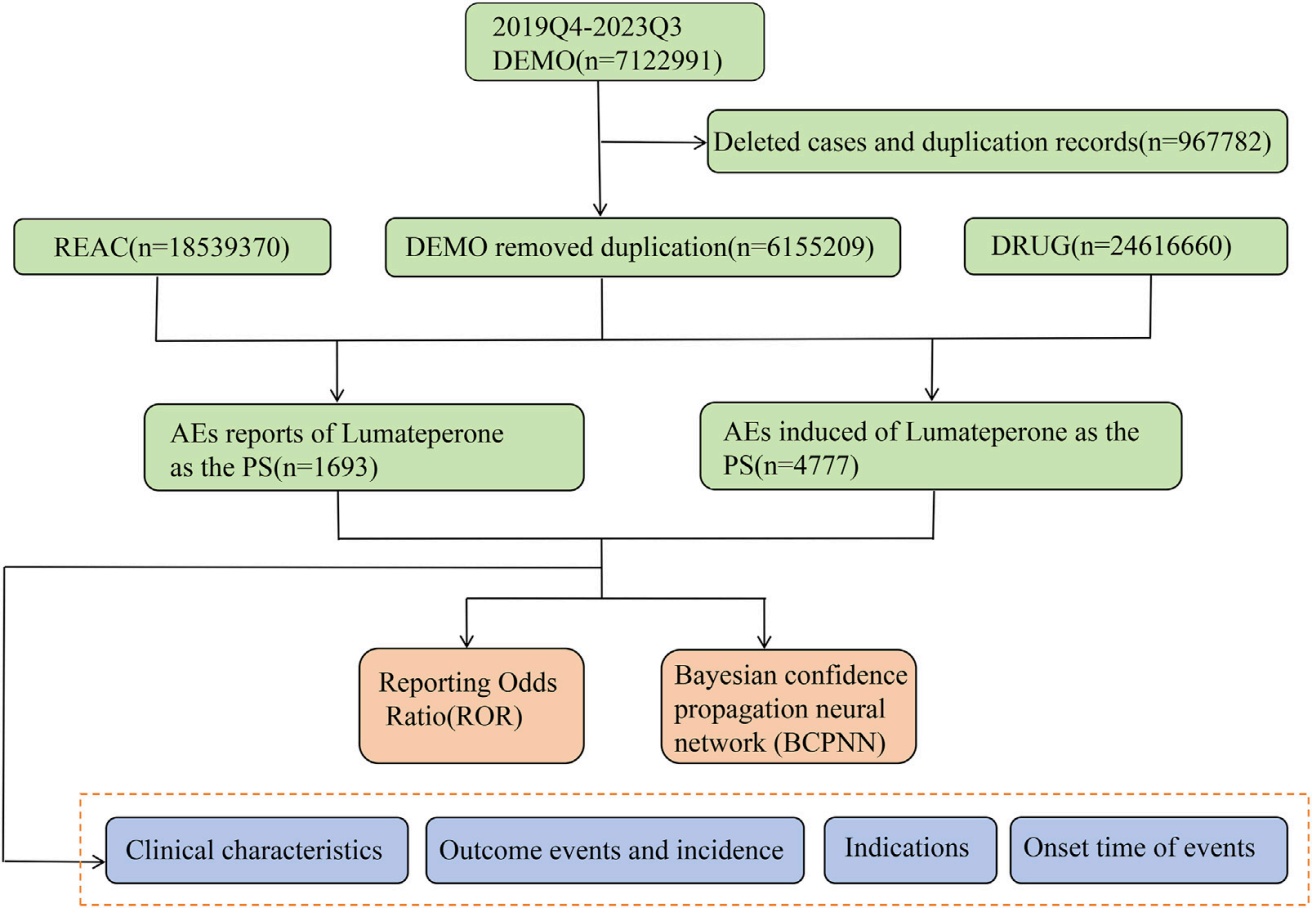
Flowchart for extracting and analyzing lumateperone-associated AEs from FAERS database.

All procedures for data manipulation and statistical evaluations were executed using R software version 4.2.3 (https://www.r-project.org/). A visual representation of the methodology of the data extraction and analysis process is shown in Figure 1.

## 3 Results

### 3.1 Description of lumateperone AEs

Within the FAERS database, a total of 1,693 patients were identified to have experienced 4,777 adverse events (AEs) attributed to lumateperone, averaging 2.8 AEs per individual. A predominant 99.9% of the patients were of United States descent (*n* = 1,692). The year 2022 saw the highest number of reports (*n* = 841), accounting for 49.7% of the reports over the recent years. Reporters were primarily health professionals, physicians, and consumers, comprising 98.2% of the total reporting figures. Age data was available for 1,487 patients (average age = 40.98 ± 14.78 years). There were 935 females (55.2%), 552 males (32.6%), and 206 instances (12.2%) where gender was unspecified. Patients under 18 years of age (*n* = 6) constituted less than 1% (0.4%), those aged 18–85 years (*n* = 623) represented 36.8%, and age information was not reported for 1,064 individuals. The most common indications were schizophrenia and bipolar disorder (including bipolar I and II), consistent with the FDA-approved indications for lumateperone.

Demographic details related to lumateperone and basic information on the adverse events are summarized in Table 1.

**TABLE 1:** Clinical characteristics of reports of lumateperone from the FAERS database (2019 quarter4 to 2023 quarter3).

Characteristics	Case number, *n*	Case proportion (%)
Number of events	1,693	
Gender		
Male	552	32.6
Unknown	206	12.2
Age		
<18	6	0.4
18–64	584	34.5
65–85	39	2.3
Unknown	1,064	62.8
Indications (TOP five)		
Bipolar disorder	520	30.7
Schizophrenia	343	20.3
Bipolar II disorder	75	4.4
Bipolar I disorder	55	3.2
Schizoaffective disorder	45	2.7
Serious outcome^a^		
Death	22	1.3
Life-Threatening	5	0.3
Hospitalization (initial or prolonged)	155	8.9
Disability	14	0.8
Required Intervention to Prevent Permanent Impairment/Damage	4	0.2
Other Serious medical events	381	21.9
Missing	1,156	66.6
Reported Countries		
United States	1,692	99.9
Poland	1	0.1
Reported Person		
Health professional	703	41.5
Consumer	598	35.3
Physician	362	21.4
Pharmacist	18	1.1
Missing	12	0.7
Reporting year		
2020	126	7.4
2021	330	19.5
2022	841	49.7
2023	396	23.4

^a^ A report may have one or more outcomes of events.

### 3.2 Signals of disproportionality in the system organ class

**FIGURE 2:**
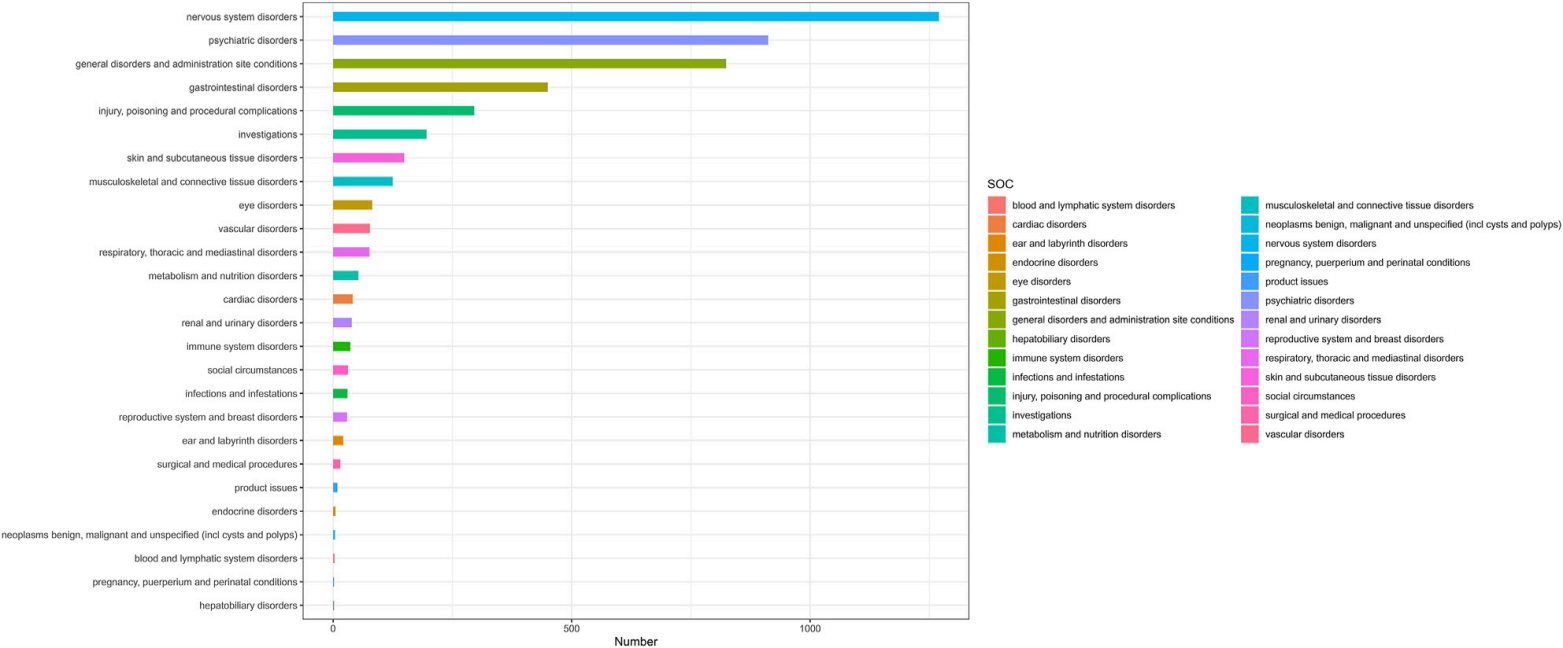
The number of lumateperone-induced AEs at the System Organ Class (SOC) level in FAERS database (n=4,777). Number, The number of AEs induced by lumateperone.

Adverse events associated with lumateperone encompassed a total of 26 organ systems (refer to Table 2; Figure 2). Significant System Organ Classes (SOCs) meeting the thresholds set by ROR or BCPNN were identified as follows: psychiatric disorders [*n* = 912, with a ROR of 4.07 and a 95% Confidence Interval (CI) of 3.79–4.38; Information Component (IC) of 1.80 and a lower bound of the 95% credibility interval (IC 025) at 0.13], nervous system disorders (*n* = 1,270, ROR = 4.62, 95% CI = 4.33–4.93; IC = 1.87, IC 025 = 0.20), and gastrointestinal system disorders (*n* = 450, ROR = 1.23, 95% CI = 1.12–1.35; IC = 0.27, IC 025 = −1.39).

**TABLE 2:** Signal strength of reports of lumateperone at the System Organ Class (SOC) level in the FAERS database.

System organ class (SOC)	Case numbers	ROR (95% Two-Sided CI)	IC (IC025)
Nervous system disorders	1,270	4.62 (4.33,4.93)*	1.87 (0.20)*
Psychiatric disorders	912	4.07 (3.79,4.38)*	1.80 (0.13)*
General disorders and administration site conditions	824	0.97 (0.90,1.04)	−0.04 (−1.70)
Gastrointestinal disorders	450	1.23 (1.12,1.35)*	0.27 (−1.39)
Injury, poisoning and procedural complications	296	0.48 (0.43,0.54)	−0.96 (−2.63)
Investigations	196	0.68 (0.59,0.79)	−0.52 (−2.19)
Skin and subcutaneous tissue disorders	149	0.56 (0.48,0.66)	−0.80 (−2.46)
Musculoskeletal and connective tissue disorders	125	0.49 (0.41,0.59)	−0.98 (−2.65)
Eye disorders	82	0.90 (0.72,1.12)	−0.15 (−1.82)
Vascular disorders	77	0.87 (0.69,1.08)	−0.20 (−1.87)
Respiratory, thoracic and mediastinal disorders	76	0.34 (0.27,0.43)	−1.50 (−3.16)
Metabolism and nutrition disorders	53	0.58 (0.44,0.76)	−0.78 (−2.45)
Cardiac disorders	41	0.43 (0.32,0.59)	−1.20 (−2.86)
Renal and urinary disorders	39	0.42 (0.31,0.57)	−1.24 (−2.90)
Immune system disorders	36	0.66 (0.47,0.91)	−0.60 (−2.26)
Social circumstances	31	1.37 (0.96,1.95)	0.45 (−1.21)
Infections and infestations	30	0.11 (0.07,0.15)	−3.15 (−4.82)
Reproductive system and breast disorders	29	0.97 (0.67,1.40)	−0.05 (−1.71)
Ear and labyrinth disorders	21	1.08 (0.70,1.65)	0.11 (−1.56)
Surgical and medical procedures	15	0.22 (0.13,0.36)	−2.18 (−3.85)
Product issues	9	0.01 (0.05,0.20)	−3.27 (−4.94)
Endocrine disorders	5	0.40 (0.17,0.97)	−1.31 (−2.97)
Neoplasms benign, malignant and unspecified (incl cysts and polyps)	4	0.02 (0.01,0.05)	−5.68 (−7.35)
Blood and lymphatic system disorders	3	0.04 (0.01,0.11)	−4.75 (−6.42)
Hepatobiliary disorders	2	0.05 (0.01,0.21)	−4.27 (−5.93)
Pregnancy, puerperium and perinatal conditions	2	0.12 (0.03,0.48)	−3.04 (−4.71)

Abbreviation: * indicate statistically significant signals in algorithm. ROR, reporting odds ratio; CI, confidence interval; IC, information component (IC); IC, 025, the lower limit of 95% CI, of the IC.

### 3.3 Preferred term signals and subgroup analyses

Employing both ROR and BCPNN methodologies, a total of 126 adverse events induced by lumateperone were identified across the 26 System Organ Classes (SOCs) involved, as shown in Supplementary Table S3. The Preferred Terms (PT) were ranked by the frequency of adverse event reporting and the strength of adverse event signals, respectively. Table 3 presents the top 20 PTs ranked by the frequency of adverse event reporting; the top 20 PTs by adverse event signal strength are shown in Table 4. Our study identified significant unexpected adverse events not mentioned in the lumateperone drug label, such as mania (ROR = 73.82, 95% CI = 57.09–95.46; IC = 6.16, IC 025 = 4.49), hypomania (ROR = 34.74, 95% CI = 15.54–77.64; IC = 5.10, IC 025 = 3.43), aggression (ROR = 10.85, 95% CI = 7.43–15.85; IC = 3.42, IC 025 = 1.76), agitation (ROR = 6.96, 95% CI = 4.77–10.17; IC = 2.79, IC 025 = 1.12), serotonin syndrome (ROR = 8.69, 95% CI = 4.81–15.72; IC = 3.11, IC 025 = 1.45), urinary retention (ROR = 3.59, 95% CI = 1.80–7.19; IC = 1.84, IC 025 = 0.18), and pseudostroke (ROR = 122.6, 95% CI = 38.83–387.08; IC = 6.89, IC 025 = 5.20).

**TABLE 3:** Top 20 AE frequency of lumateperone.

SOC name	Preferred terms (PTs)	*n*	ROR (95% two sided CI)	IC (IC025)
Nervous system disorders	Dizziness^a^	215	6.77 (5.91,7.77)	2.7 (1.04)
Nervous system disorders	Somnolence^a^	123	9.23 (7.71,11.04)	3.17 (1.50)
General disorders and administration site conditions	Feeling abnormal	103	6.30 (5.18,7.66)	2.63 (0.96)
Nervous system disorders	Sedation^a^	76	44.51 (35.44,55.91)	5.44 (3.77)
Psychiatric disorders	Insomnia	69	4.16 (3.28,5.28)	2.04 (0.37)
Nervous system disorders	Burning sensation^b^	68	15.38 (12.10,19.55)	3.92 (2.25)
Psychiatric disorders	Mania	60	73.82 (57.09,95.46)	6.16 (4.49)
Gastrointestinal disorders	Dry mouth^a^	58	11.59 (8.94,15.02)	3.52 (1.85)
Psychiatric disorders	Suicidal ideation^c^	56	10.94 (8.40,14.24)	3.43 (1.77)
Nervous system disorders	Tardive dyskinesia^c^	54	66.92 (51.06,87.71)	6.02 (4.36)
General disorders and administration site conditions	Unevaluable event	49	8.97 (6.77,11.89)	3.15 (1.48)
Psychiatric disorders	Psychotic disorder	41	25.40 (18.66,34.58)	4.65 (2.98)
General disorders and administration site conditions	Performance status decreased	39	167.28 (121.26,230.77)	7.31 (5.65)
Psychiatric disorders	Hallucination, auditory	38	37.91 (27.50,52.24)	5.22 (3.55)
Nervous system disorders	Paraesthesia	34	3.23 (2.31,4.53)	1.68 (0.02)
Nervous system disorders	Tremor	34	3.30 (2.36,4.63)	1.72 (0.05)
Skin and subcutaneous tissue disorders	Skin burning sensation^b^	34	5.65 (4.03,7.92)	2.49 (0.82)
Nervous system disorders	Migraine	32	4.32 (3.05,6.11)	2.1 (0.44)
General disorders and administration site conditions	Feeling hot	31	7.27 (5.10,10.35)	2.85 (1.18)
Psychiatric disorders	Hallucination	28	5.08 (3.50,7.36)	2.34 (0.67)

Abbreviation: *n*, Number of cases reporting PT; ROR, reporting odds ratio; CI, confidence interval; IC, information component; IC, 025, the lower 95% CI, of IC.

^a^ The PTs, listed were existing in the Clinical Trials Experience.

^b^ The PTs, listed were existing in the Postmarketing Experience.

^c^ The PTs, listed were existing in the warning section of the drug label.

**TABLE 4:** Top 20 signal strength of AEs of lumateperone.

SOC name	Preferred terms (PTs)	*n*	ROR (95% two sided CI)	IC (IC025)
Psychiatric disorders	Trance	3	176.47 (55.47,561.47)	7.40 (5.70)
General disorders and administration site conditions	Performance status decreased	39	167.28 (121.26,230.77)	7.31 (5.65)
Psychiatric disorders	Schizoaffective disorder bipolar type	3	135.43 (42.82,428.37)	7.03 (5.34)
Nervous system disorders	Pseudostroke	3	122.60 (38.83,387.08)	6.89 (5.20)
Psychiatric disorders	Mania	60	73.82 (57.09,95.46)	6.16 (4.49)
Nervous system disorders	Tardive dyskinesia^a^	54	66.92 (51.06,87.71)	6.02 (4.36)
Psychiatric disorders	Soliloquy	4	65.82 (24.49,176.89)	6.02 (4.34)
General disorders and administration site conditions	Temperature regulation disorder	12	61.82 (34.93,109.42)	5.92 (4.25)
Gastrointestinal disorders	Tongue movement disturbance	5	60.50 (25.00,146.41)	5.90 (4.22)
General disorders and administration site conditions	Hangover	11	52.29 (28.83,94.87)	5.69 (4.02)
Psychiatric disorders	Autoscopy	3	48.73 (15.60,152.23)	5.59 (3.91)
Nervous system disorders	Sedation^b^	76	44.51 (35.44,55.91)	5.44 (3.77)
General disorders and administration site conditions	Feeling drunk	16	42.84 (26.15,70.17)	5.40 (3.73)
Psychiatric disorders	Hallucination, auditory	38	37.91 (27.50,52.24)	5.22 (3.55)
Psychiatric disorders	Hypomania	6	34.74 (15.54,77.64)	5.10 (3.43)
Nervous system disorders	Sleep paralysis	3	31.06 (9.97,96.77)	4.94 (3.27)
Nervous system disorders	Neuroleptic malignant syndrome^a^	19	30.69 (19.53,48.25)	4.92 (3.26)
Psychiatric disorders	Paranoia	27	29.43 (20.13,43.02)	4.86 (3.19)
Psychiatric disorders	Intrusive thoughts	3	27.02 (8.68,84.15)	4.75 (3.07)
Nervous system disorders	Akathisia	22	26.59 (17.47,40.49)	4.72 (3.05)

Abbreviation: *n*, Number of cases reporting PT; ROR, reporting odds ratio; CI, confidence interval; IC, information component; IC, 025, the lower 95% CI, of IC.

^a^ The PTs, listed were existing in the warning section of the drug label.

^b^ The PTs, listed were existing in the Clinical Trials Experience.

Subgroup analyses were performed for gender, calculating adverse event signals for males and females (Supplementary Tables S4A, B). The top 10 reported AEs in each subgroup were analyzed. It was found that only the male subgroup reported “headache” and “nausea,” while “burning sensation” and “delayed onset movement disorders” appeared to be more common in the female subgroup.

### 3.4 Comparision with risperidone

We chose risperidone to conduct a comparative disproportionality analysis on a set of adverse events associated with lumateperone: serotonin syndrome, urinary retention, and pseudostroke. As of the third quarter of 2023, no reports of pseudostroke caused by risperidone were found in the FAERS database. The FAERS database registered 177 reports of serotonin syndrome related to risperidone, with 4,766 and 214,937 other adverse drug events reported with lumateperone and risperidone, respectively. The comparative OR of serotonin syndrome with lumateperone was 2.80 (95% CI: 1.52–5.16).

Likewise, 200 reports of urinary retention were registered with lumateperone. There were 4,769 and 214,914 other adverse drug event reports related to lumateperone and risperidone, respectively. The comparative OR of urinary retention with lumateperone was 1.80 (95% CI: 0.89–3.66).

### 3.5 Time-to-onset analysis

**FIGURE 3:**
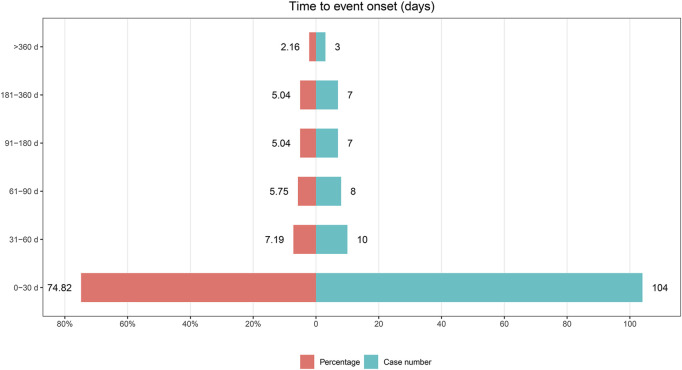
Time to onset of lumateperone-related AEs.

Among the 1,693 lumateperone-related reports analyzed, data on the timing of adverse event onset was available in 139 reports. The analysis of the onset timing for these adverse events, along with Weibull distribution test results, are detailed in Table 5. The median time to onset of lumateperone-associated adverse events was identified as 13 days, with a range of 2–30 days. The Weibull Shape Parameter (WSP) test for the onset timing of these adverse events demonstrated that the upper limit of the 95% confidence interval (CI) for the shape parameter was less than 1, suggesting a tendency towards early failure. Furthermore, the distribution of adverse event onset times, illustrated in Figure 3, indicated that 74.82% of adverse events (*n* = 104) occurred within the first month of lumateperone administration, with a subsequent consistent decrease in the number of adverse events from the second month onward.

**TABLE 5:** Time-to-onset analysis for signals with lumateperone.

Drug	TTO (days)		Weibull distribution		
	Case reports(n)	Median(d) (IQR)	Scale parameter:α (95% CI)	Shape parameter:β (95% CI)	Type
Lumateperone	139	13 (2–30)	25.61 (17.95–33.28)	0.59 (0.52,0.66)	Early failure

Abbreviation: TTO, Time-to-onset; n, number of cases with available time-to-onset; IQR, interquartile range; CI, confidence interval.

## 4 Discussion

This pharmacovigilance study represents the most comprehensive and systematic real-world investigation into the safety profile of lumateperone based on the FAERS database to date. Our findings identified important adverse events not listed on the lumateperone drug label, raising pertinent questions regarding the efficacy and safety of lumateperone. Collectively, our analysis underscores the importance of ongoing surveillance of lumateperone’s safety and efficacy through more precise and thorough real-world evaluations. This endeavor aims to enrich clinical decision-making with further evidence and novel insights, emphasizing that continuous monitoring plays a critical role in ensuring the therapeutic’s safety profile.

Lumateperone significantly ameliorates positive and negative symptoms of schizophrenia as well as cognitive impairments (Correll et al., 2020). Additionally, it notably alleviates depressive symptoms and reduces the severity of bipolar disorder (McIntyre et al., 2023). However, with the widespread use of lumateperone, an increasing number of adverse events have been observed in the real world. Therefore, conducting pharmacovigilance analysis is vital for investigating the safety profile of lumateperone. We conducted disproportionality analysis using the FAERS database and identified frequently occurring safety signals such as somnolence, sedation, and dry mouth—adverse events that are documented within the drug’s label and reported in the relevant literature (Calabrese et al., 2021; Kane et al., 2021; Maini et al., 2021). Moreover, we uncovered several adverse events not previously reported in regulatory trials, including mania, hypomania, suicidal ideation, homicidal ideation, migraines, urinary retention, and sexual dysfunction. Overall, through the analysis of demographic characteristics of 1,693 patients and lumateperone-related safety signals, our investigation yielded five primary findings.

Firstly, our study found that both mania and hypomania exhibited higher signal strengths in the disproportionality analysis. This suggests the potential of lumateperone to induce mania and hypomania, especially in the therapeutic context of depressive episodes in bipolar disorder. This significant finding indicates a potential risk of phase shift in patients after taking lumateperone, a critical aspect in the management of bipolar disorder. Typically, the treatment goal for bipolar disorder is to achieve enhanced mood stability without triggering a bipolar shift in mood (Malhi et al., 2015). Therefore, in patients treated with lumateperone, especially those with bipolar depression episodes, the emergence of symptoms such as insomnia and agitation warrants a careful reevaluation of its use in this cohort. The induction of elevated mood symptoms could be attributed to the modulation of neurotransmitters associated with lumateperone, particularly its high affinity for 5-HT2A or the elevated 5-HT2A/D2 ratio (Lu et al., 2002; Privitera and Maharaj, 2003; Khalil and Baddoura, 2012). Additionally, the concurrent use of other medications, age at first episode, and family history are also contributing factors to the induction of mania or hypomania (Ramasubbu, 2001; Amsterdam et al., 2004). Clinicians prescribing lumateperone should exercise increased vigilance and conduct a comprehensive assessment, especially in patients with bipolar disorder.

The second aspect is that our study identified rare serious adverse events of lumateperone, such as urinary retention, serotonin syndrome, and pseudostroke. Urinary retention is a distressing condition that requires repeated indwelling catheters, which increase the risk of urinary tract infections (Bozikas et al., 2001). Its occurrence raises concerns about the potential anticholinergic effects of lumateperone, a common but often neglected side effect of many psychotropic medications (Faure Walker et al., 2016). Serotonin syndrome is a potentially life-threatening condition resulting from excessive serotonergic activity in the central nervous system by mechanisms that may involve 5-HT1A receptors, 5-HT2 receptors, and 5-HT3 receptors (Binienda et al., 2018; Scotton et al., 2019). This signal also stands out remarkably when lumateperone is compared to risperidone. The concurrent use of risperidone with antidepressants is prone to causing serotonin syndrome (Direk et al., 2016; Kohen et al., 2007). Pseudostrokes exhibit stroke-like symptoms that are not attributable to an actual cerebrovascular event (Behrouz and Benbadis, 2014; Deneux et al., 2017). This suggests that lumateperone may have neurovascular effects that warrant further investigation. The discovery of serious adverse events associated with lumateperone suggests the necessity for further research into its pharmacological mechanisms and a reassessment of its risk-benefit profile. For the prevention of grave consequences, medical practitioners need to remain alert to these serious adverse events to ensure that they are recognized and intervened expeditiously.

Regarding the third aspect, our findings elucidate that the primary indications for lumateperone encompass schizophrenia as well as severe depressive episodes in Bipolar I and II disorders, aligning with the FDA-approved indications for the medication. However, our disproportionality analysis uncovered several safety signals related to these indications, such as psychotic disorder, hallucination, delusion, paranoia, apathy, psychotic symptoms, schizophrenia, and bipolar disorder, emotional disorder, mood swings. This observation introduces a subtle complication into the clinical understanding of the safety paradigm of lumateperone, necessitating an in-depth examination of its pharmacological implications and the accuracy of its reporting in a real-world setting. The emergence of these safety signals can be attributed to two primary factors. Firstly, the specific pharmacodynamic properties of lumateperone, particularly its role as a 5-HT2A receptor antagonist and indirect modulator of glutamatergic phosphoproteins, may lead to its overregulation of serotonergic and glutamatergic activities. Although this mechanism facilitates the alleviation of depressive symptoms, it may inadvertently lead to mood swings and may even induce mania or hypomania, with mania and hypomania having a high signal and reporting frequency in our disambiguation analyses. The pharmacological effect reflects the complexity of lumateperone’s involvement in the modulation of multiple neurotransmitter systems. Lumateperone may potentially exacerbate emotional instability, a concern that warrants attention despite the current underreporting in the literature (McIntyre et al., 2023). Whether novel antipsychotics can be used as mood stabilizers throughout the full course of bipolar disorder still needs to be explored in further studies (Zarate, 2000; Popovic et al., 2012; Pompili et al., 2018). Additionally, the potential biases inherent in the FAERS database, including both reporting bias and indication bias, highlight the challenge of distinguishing drug-induced adverse events from underlying disease symptoms (Gastaldon et al., 2021). Such discrepancies emphasize the critical need for rigorous interpretation of the data.

In the fourth aspect of our discussion, the disambiguation analyses revealed pronounced safety signals for suicidal ideation, homicidal ideation, and self-injurious ideation associated with lumateperone use, yet no similar signals were detected for actual suicide attempts or completed suicides. Our findings suggest that lumateperone may increase self-injury, suicidal ideation, and homicidal ideation, but does not increase the risk of suicidal behavior and suicide deaths, although the drug’s black box warning pertaining to suicidal behavior. We must acknowledge that the outcomes of our research might be affected by the major underreporting of adverse events, a fundamental limitation inherent to pharmacovigilance studies. Furthermore, since lumateperone is a new drug, we cannot exclude the possibility that its prolonged use may lead to risks of suicidal behavior and suicide deaths. The emergence of adverse events such as self-injurious ideation, suicidal ideation, or homicidal ideation may be associated with lumateperone’s involvement in the regulation of the serotonin neurotransmitter system, Research indicates that serotonin (5-HT) is involved in the formation of the 5-HTDRN→CRFBNST circuit, contributing to anxiety and fear during the initial phase of treatment, which may be one of the mechanisms underlying the emergence of early adverse events (Marcinkiewcz et al., 2016). However, studies have shown that with the improvement of psychotic symptoms by antipsychotic drugs, especially in terms of cognition and emotion, the risk of suicide and aggression will ultimately be reduced (Meltzer and Gadaleta, 2021). Additionally, suicidal behaviors and suicide deaths are influenced by multiple factors, such as disease progression, awareness of symptoms, and comorbid depression (Sher and Kahn, 2019; Keramatian et al., 2023). Thus, the relationship between antipsychotics and suicide is complex and controversial, and the therapeutic benefits and potential risks of psychotropic medications need to be carefully balanced.

The application of Weibull distribution analysis in pharmacovigilance offers a robust method for assessing the likelihood and timing of drug-related adverse events (Mazhar et al., 2021). Our analysis revealed an early failure pattern, indicating an increased propensity for adverse events shortly after treatment initiation, rather than a uniform distribution or an increase over time. Lumateperone-related adverse events predominantly occur within the first month of use, with a median onset time of 13 days. Therefore, vigilant monitoring and proactive management of adverse events associated with lumateperone, especially during the initial stages of treatment, are essential. Analysis of the annual variation in lumateperone reporting showed an incremental increase from 2019 to 2021, with the peak number of reports in 2021, followed by a decrease in 2022, returning to the levels seen in 2020. This trend aligns with the Weber effect, where a peak in reporting occurs 2 years after drug approval (Hoffman et al., 2014). Consequently, a future decline in lumateperone report volumes may be anticipated. Nonetheless, the limited extensive use of lumateperone across various countries and potential under-recognition due to its recent market introduction might also lead to a reduction in reporting volumes. Thus, continued concern for future reports and the necessity for ongoing epidemiological surveillance remain paramount.

There are several limitations in our research. Firstly, the FAERS database has potential biases, such as reporting bias and indication bias, It may be challenging to determine whether an adverse event is induced by the drug or is an exacerbation of the disease itself. Moreover, we are also unable to obtain precise incidence rates or establish a definitive causal relationship between drug exposure and adverse events. Therefore, future epidemiological research is necessary to further clarify these associations. Secondly, the database fundamentally relies on spontaneous reporting, and the voluntary nature of the system often leads to incomplete data submissions, complicating the robustness and reliability of the captured adverse event information. This incompleteness can hinder the ability to draw firm conclusions about drug safety profiles. Finally, the disproportionality analysis method does not completely eliminate the confounding effect of combined medications. Nevertheless, leveraging the FAERS database for pharmacovigilance research offers the advantage of accessing a vast, real-world dataset, enabling the early identification of drug safety signals and trends across a diverse population. This approach significantly enhances our capacity to monitor post-marketing drug safety and implement timely interventions to protect public health.

## 5 Conclusion

In conclusion, the findings of this study suggest the potential for lumateperone to induce mania and trigger certain rare, severe adverse events, which may reignite concerns regarding its safety and efficacy profile. Healthcare providers should exercise vigilance in monitoring patients undergoing lumateperone treatment. Ongoing pharmacovigilance and epidemiological research, along with prudent risk-benefit assessments, are crucial for further elucidating the risks associated with lumateperone and ensuring patient safety.

## Data Availability

The datasets presented in this study can be found in online repositories. The names of the repository/repositories and accession number(s) can be found below: https://fis.fda.gov/extensions/FPD-QDE-FAERS/FPD-QDE-FAERS.html.

## References

[B1] Abuelazm H. Elsayed O. H. El-Mallakh R. S. (2023). Evaluating lumateperone for its use in treating depressive episodes associated with bipolar I or II disorder in adults. Expert. Rev. Neurother. 23, 751–756. 10.1080/14737175.2023.2236795 37458003

[B2] Amsterdam J. D. Shults J. Brunswick D. J. Hundert M. (2004). Short-term fluoxetine monotherapy for bipolar type II or bipolar NOS major depression - low manic switch rate. Bipolar. Disord. 6, 75–81. 10.1046/j.1399-5618.2003.00083.x 14996144

[B3] Bate A. Lindquist M. Edwards I. R. Olsson S. Orre R. Lansner A. (1998). A Bayesian neural network method for adverse drug reaction signal generation. Eur. J. Clin. Pharmacol. 54, 315–321. 10.1007/s002280050466 9696956

[B4] Behrouz R. Benbadis S. R. (2014). Psychogenic pseudostroke. J. Stroke. Cerebrovasc. Dis. 23, e243–e248. 10.1016/j.jstrokecerebrovasdis.2013.11.010 24439129

[B5] Binienda A. Storr M. Fichna J. Salaga M. (2018). Efficacy and safety of serotonin receptor ligands in the treatment of irritable bowel syndrome: a review. Curr. Drug. Targets 19, 1774–1781. 10.2174/1389450119666171227225408 29284389

[B6] Blair H. A. (2020). Lumateperone: first approval. Drugs 80, 417–423. 10.1007/s40265-020-01271-6 32060882

[B7] Bozikas V. Petrikis P. Karavatos A. (2001). Urinary retention caused after fluoxetine-risperidone combination. J. Psychopharmacol. 15, 142–143. 10.1177/026988110101500201 11448089

[B8] Calabrese J. R. Durgam S. Satlin A. Vanover K. E. Davis R. E. Chen R. (2021). Efficacy and safety of lumateperone for major depressive episodes associated with bipolar I or bipolar II disorder: a phase 3 randomized placebo-controlled trial. Am. J. Psychiatry. 178, 1098–1106. 10.1176/appi.ajp.2021.20091339 34551584

[B9] Caster O. Aoki Y. Gattepaille L. M. Grundmark B. (2020). Disproportionality analysis for pharmacovigilance signal detection in small databases or subsets: recommendations for limiting false-positive associations. Drug Saf. 43, 479–487. 10.1007/s40264-020-00911-w 32008183 PMC7165139

[B10] Chavant F. Favrelière S. Lafay-Chebassier C. Plazanet C. Perault-Pochat M. C. (2011). Memory disorders associated with consumption of drugs: updating through a case/noncase study in the French pharmacovigilance database. Br. J. Clin. Pharmacol. 72, 898–904. 10.1111/j.1365-2125.2011.04009.x 21557759 PMC3244635

[B11] Chrétien B. Lelong-Boulouard V. Chantepie S. Sassier M. Bertho M. Brazo P. (2021). Haematologic malignancies associated with clozapine v. all other antipsychotic agents: a pharmacovigilance study in VigiBase^®^ . Psychol. Med. 51, 1459–1466. 10.1017/S0033291720000161 32036793

[B12] Correll C. U. Davis R. E. Weingart M. Saillard J. O'Gorman C. Kane J. M. (2020). Efficacy and safety of lumateperone for treatment of schizophrenia: a randomized clinical trial. JAMA Psychiatry 77, 349–358. 10.1001/jamapsychiatry.2019.4379 31913424 PMC6990963

[B13] Deneux V. Leboucq N. Saumet L. Haouy S. Akbaraly T. Sirvent N. (2017). Acute methotrexate-related neurotoxicity and pseudo-stroke syndrome. Arch. Pediatr. 24, 1244–1248. 10.1016/j.arcped.2017.09.024 29146215

[B14] Direk M. Ç. Yıldırım V. Güneş S. Bozlu G. Okuyaz Ç. (2016). Serotonin syndrome after clomipramine overdose in a child. Clin. Psychopharmacol. Neurosci. 14, 388–390. 10.9758/cpn.2016.14.4.388 27776393 PMC5083934

[B15] Durgam S. Satlin A. Davis R. E. Vanover K. E. Mates S. Kane J. M. (2020). T205. Lumateperone in the treatment of schizophrenia: evaluation of extrapyramidal and motor symptoms in 4 late-phase clinical trials. Schizophr. Bull. 46 (Suppl. l), S310. 10.1093/schbul/sbaa029.765

[B16] Edinoff A. Wu N. Deboisblanc C. Feltner C. O. Norder M. Tzoneva V. (2020). Lumateperone for the treatment of schizophrenia. Psychopharmacol. Bull. 50, 32–59. 33012872 10.64719/pb.4372 PMC7511146

[B17] Faure Walker N. Brinchmann K. Batura D. (2016). Linking the evidence between urinary retention and antipsychotic or antidepressant drugs: a systematic review. Neurourol. Urodyn. 35, 866–874. 10.1002/nau.22851 26288221

[B18] Ford I. Norrie J. (2016). Pragmatic trials. N. Engl. J. Med. 375, 454–463. 10.1056/NEJMra1510059 27518663

[B19] Gastaldon C. Raschi E. Kane J. M. Barbui C. Schoretsanitis G. (2021). Post-marketing safety concerns with esketamine: a disproportionality analysis of spontaneous reports submitted to the FDA adverse event reporting system. Psychother. Psychosom. 90, 41–48. 10.1159/000510703 32854103

[B20] Hoffman K. B. Dimbil M. Erdman C. B. Tatonetti N. P. Overstreet B. M. (2014). The weber effect and the United States food and drug administration's adverse event reporting system (FAERS): analysis of sixty-two drugs approved from 2006 to 2010. Drug Saf. 37, 283–294. 10.1007/s40264-014-0150-2 24643967 PMC3975089

[B21] Jawwad M. Y. Alnefeesi Y. Ceban F. Lui L. M. W. Jaberi S. Di Vincenzo J. D. (2022). Lumateperone for the treatment of adults with schizophrenia: a systematic review. Curr. Psychiatry. Rep. 24, 359–368. 10.1007/s11920-022-01344-1 35802228

[B22] Jiang Y. Zhou L. Y. Shen Y. Zhou Q. Ji Y. Y. Zhu H. H. (2024). Safety assessment of brexpiprazole: real-world adverse event analysis from the FAERS database. J. Affect. Disord. 346, 223–229. 10.1016/j.jad.2023.11.025 37956832

[B23] Kane J. M. Durgam S. Satlin A. Vanover K. E. Chen R. Davis R. (2021). Safety and tolerability of lumateperone for the treatment of schizophrenia: a pooled analysis of late-phase placebo- and active-controlled clinical trials. Int. Clin. Psychopharmacol. 36, 244–250. 10.1097/YIC.0000000000000371 34054112 PMC8322041

[B24] Keramatian K. Chakrabarty T. Dubow A. Saraf G. Yatham L. N. (2023). New pharmacologic approaches to the treatment of bipolar depression. Drugs 83, 843–863. 10.1007/s40265-023-01872-x 37227597

[B25] Khalil R. B. Baddoura C. (2012). Quetiapine induced hypomania: a case report and a review of the literature. Curr. Drug Saf. 7, 250–253. 10.2174/157488612803251333 22950378

[B26] Kinoshita S. Hosomi K. Yokoyama S. Takada M. (2020). Time-to-onset analysis of amiodarone-associated thyroid dysfunction. J. Clin. Pharm. Ther. 45, 65–71. 10.1111/jcpt.13024 31400296

[B27] Kohen I. Gordon M. L. Manu P. (2007). Serotonin syndrome in elderly patients treated for psychotic depression with atypical antipsychotics and antidepressants: two case reports. CNS Spectr. 12, 596–598. 10.1017/s1092852900021386 17667887

[B28] Kumar B. Kuhad A. Kuhad A. (2018). Lumateperone: a new treatment approach for neuropsychiatric disorders. Drugs Today(Barc) 54, 713–719. 10.1358/dot.2018.54.12.2899443 30596390

[B29] Lu B. Y. Lundgren R. Escalona P. R. Roberts B. B. (2002). A case of ziprasidone-induced mania and the role of 5-HT2A in mood changes induced by atypical antipsychotics. J. Clin. Psychiatry. 63, 1185–1186. 10.4088/jcp.v63n1214j 12530423

[B30] Maini K. Hollier J. W. Gould H. Bollich V. John-LaForge J. Cornett E. M. (2021). Lumateperone tosylate, a selective and concurrent modulator of serotonin, dopamine, and glutamate, in the treatment of schizophrenia. Health Psychol. Res. 9, 24932. 10.52965/001c.24932 34746489 PMC8567771

[B31] Malhi G. S. McAulay C. Das P. Fritz K. (2015). Maintaining mood stability in bipolar disorder: a clinical perspective on pharmacotherapy. Evid. Based Ment. Health 18, 1–6. 10.1136/eb-2014-101948 25165167 PMC11235049

[bib46] Marcinkiewcz C. A. Mazzone C. M. D’Agostino G. Halladay L. R. Hardaway J. A. DiBerto J. F. (2016). Serotonin engages an anxiety and fear-promoting circuit in the extended amygdala. Nature 537, 97–101. 10.1038/nature19318 27556938 PMC5124365

[B32] Mazhar F. Battini V. Gringeri M. Pozzi M. Mosini G. Marran A. M. N. (2021). The impact of anti-TNFα agents on weight-related changes: new insights from a real-world pharmacovigilance study using the FDA adverse event reporting system (FAERS) database. Expert Opin. Biol. Ther. 21, 1281–1290. 10.1080/14712598.2021.1948529 34191656

[B33] McIntyre R. S. Durgam S. Huo J. Kozauer S. G. Stahl S. M. (2023). The efficacy of lumateperone in patients with bipolar depression with mixed features. J. Clin. Psychiatry. 84, 22m14739. 10.4088/JCP.22m14739 37103915

[B34] Meltzer H. Y. Gadaleta E. (2021). Contrasting typical and atypical antipsychotic drugs. Focus Am Psychiatr. Publ. 19, 3–13. 10.1176/appi.focus.20200051 34483761 PMC8412155

[B35] Noguchi Y. Tachi T. Teramachi H. (2021). Detection algorithms and attentive points of safety signal using spontaneous reporting systems as a clinical data source. Brief. Bioinform. 22, bbab347. 10.1093/bib/bbab347 34453158

[B36] Pompili M. Verzura C. Trovini G. Buscajoni A. Falcone G. Naim S. (2018). Lurasidone: efficacy and safety in the treatment of psychotic and mood disorders. Expert Opin. Drug Saf. 17, 197–205. 10.1080/14740338.2017.1379989 28902525

[B37] Popovic D. Reinares M. Goikolea J. M. Bonnin C. M. Gonzalez-Pinto A. Vieta E. (2012). Polarity index of pharmacological agents used for maintenance treatment of bipolar disorder. Eur. Neuropsychopharmacol. 22, 339–346. 10.1016/j.euroneuro.2011.09.008 22000157

[B38] Privitera M. R. Maharaj K. (2003). Mania from dose-related ziprasidone augmentation of an SSRI. J. Clin. Psychiatry. 64, 1392–1394. 10.4088/jcp.v64n1116d 14658958

[B39] Ramasubbu R. (2001). Dose-response relationship of selective serotonin reuptake inhibitors treatment-emergent hypomania in depressive disorders. Acta. Psychiatr. Scand. 104, 236–238. 10.1034/j.1600-0447.2001.00383.x 11531662

[B40] Raschi E. Mazzarella A. Antonazzo I. C. Bendinelli N. Forcesi E. Tuccori M. (2019). Toxicities with immune checkpoint inhibitors: emerging priorities from disproportionality analysis of the FDA adverse event reporting system. Target. Oncol. 14, 205–221. 10.1007/s11523-019-00632-w 30927173

[B41] Sakaeda T. Tamon A. Kadoyama K. Okuno Y. (2013). Data mining of the public version of the FDA adverse event reporting system. Int. J. Med. Sci. 10, 796–803. 10.7150/ijms.6048 23794943 PMC3689877

[B42] Scotton W. J. Hill L. J. Williams A. C. Barnes N. M. (2019). Serotonin syndrome: pathophysiology, clinical features, management, and potential future directions. Int. J. Tryptophan Res. 12, 1178646919873925. 10.1177/1178646919873925 31523132 PMC6734608

[B43] Sher L. Kahn R. S. (2019). Suicide in schizophrenia: an educational overview. Med. Kaunas. 55, 361. 10.3390/medicina55070361 PMC6681260 31295938

[B44] Zarate C. A. (2000). Antipsychotic drug side effect issues in bipolar manic patients. J. Clin. Psychiatry. 61 (8), 52–63. 10811244

[B45] Zou F. Zhu C. Lou S. Cui Z. Wang D. Ou Y. (2023). A real-world pharmacovigilance study of mepolizumab in the FDA adverse event reporting system (FAERS) database. Front. Pharmacol. 14, 1320458. 10.3389/fphar.2023.1320458 38186645 PMC10771301

