# Supplementary material for: Post-marketing safety concerns with lumateperone: a pharmacovigilance analysis based on the FDA adverse event reporting system (FAERS) database
Source: Front Pharmacol. 2024 May 9;15:1389814. doi: 10.3389/fphar.2024.1389814 (PMC11111848; doi:10.3389/fphar.2024.1389814)
Supplement: Supplementary file 1 [file Table1.DOCX]

Supplementary Material

Supplementary Table 1:Two-by-two contingency table for disproportionality analyses.

|  | Target AE | Other AEs | Total |
| --- | --- | --- | --- |
| Target drug | a | b | a+b |
| Other drugs | c | d | c+d |
| Total | a+c | b+d | a+b+c+d |

Abbreviation: AE, adverse event.

Supplementary table 2. The specific formulas for the two algorithms are as follows.

| Algorithms | Equation | Criteria |
| --- | --- | --- |
| ROR | ROR=($a$/$c$)/($b$/$d$)=$\mathrm{ad}$/$\mathrm{bc}$ | a ≥ 3,  Lower limit of 95% CI > 1, |
|  | 95%CI=$e^{lnROR\pm1.96\sqrt{(\frac{1}{a}+\frac{1}{b}+\frac{1}{c}+\frac{1}{d})}}$ |  |
| BCPNN | IC = log2a(a+b+c+d)/[(a+b)(a+c)] | IC 025 > 0 |
|  | 95%CI = E(IC) ± 2[V(IC)]^0.5 |  |

Abbreviation: ROR, reporting odds ratio; BCPNN, confidence propagation neural network; 95% CI, 95% confidence interval; N, the number of reports; IC, information component; IC025, the lower limit of the 95% CI of the IC; E (IC), the IC expectations; V (IC), the variance of IC.

Supplementary table 3. Safety signals of lumateperone compared with all other drugs.

| SOC Name | Preferred terms (PTs) | n | ROR(95% Two Sided CI) | IC(IC025) |
| --- | --- | --- | --- | --- |
| Nervous system disorders | Dizziness^a^ | 215 | 6.77(5.91,7.77) | 2.7(1.04) |
| Nervous system disorders | Somnolence^a^ | 123 | 9.23(7.71,11.04) | 3.17(1.5) |
| General disorders and administration site conditions | Feeling abnormal | 103 | 6.3(5.18,7.66) | 2.63(0.96) |
| Nervous system disorders | Sedation^a^ | 76 | 44.51(35.44,55.91) | 5.44(3.77) |
| Psychiatric disorders | Insomnia | 69 | 4.16(3.28,5.28) | 2.04(0.37) |
| Nervous system disorders | Burning sensation^b^ | 68 | 15.38(12.1,19.55) | 3.92(2.25) |
| Psychiatric disorders | Mania | 60 | 73.82(57.09,95.46) | 6.16(4.49) |
| Gastrointestinal disorders | Dry mouth^a^ | 58 | 11.59(8.94,15.02) | 3.52(1.85) |
| Psychiatric disorders | Suicidal ideation^c^ | 56 | 10.94(8.4,14.24) | 3.43(1.77) |
| Nervous system disorders | Tardive dyskinesia^c^ | 54 | 66.92(51.06,87.71) | 6.02(4.36) |
| General disorders and administration site conditions | Unevaluable event | 49 | 8.97(6.77,11.89) | 3.15(1.48) |
| Psychiatric disorders | Psychotic disorder | 41 | 25.4(18.66,34.58) | 4.65(2.98) |
| General disorders and administration site conditions | Performance status decreased | 39 | 167.28(121.26,230.77) | 7.31(5.65) |
| Psychiatric disorders | Hallucination, auditory | 38 | 37.91(27.5,52.24) | 5.22(3.55) |
| Nervous system disorders | Paraesthesia | 34 | 3.23(2.31,4.53) | 1.68(0.02) |
| Nervous system disorders | Tremor | 34 | 3.3(2.36,4.63) | 1.72(0.05) |
| Skin and subcutaneous tissue disorders | Skin burning sensation^b^ | 34 | 5.65(4.03,7.92) | 2.49(0.82) |
| Nervous system disorders | Migraine | 32 | 4.32(3.05,6.11) | 2.1(0.44) |
| General disorders and administration site conditions | Feeling hot | 31 | 7.27(5.1,10.35) | 2.85(1.18) |
| Psychiatric disorders | Hallucination | 28 | 5.08(3.5,7.36) | 2.34(0.67) |
| Psychiatric disorders | Paranoia | 27 | 29.43(20.13,43.02) | 4.86(3.19) |
| Psychiatric disorders | Agitation | 27 | 6.96(4.77,10.17) | 2.79(1.12) |
| Psychiatric disorders | Aggression | 27 | 10.85(7.43,15.85) | 3.43(1.76) |
| Nervous system disorders | Hypersomnia | 26 | 12.84(8.73,18.9) | 3.67(2) |
| Nervous system disorders | Balance disorder | 24 | 4.1(2.74,6.12) | 2.03(0.36) |
| Nervous system disorders | Akathisia | 22 | 26.59(17.47,40.49) | 4.72(3.05) |
| Nervous system disorders | Disturbance in attention | 20 | 5.88(3.79,9.13) | 2.55(0.88) |
| Gastrointestinal disorders | Dysphagia^c^ | 20 | 3.41(2.2,5.29) | 1.76(0.1) |
| General disorders and administration site conditions | Feeling cold | 20 | 10.5(6.76,16.3) | 3.38(1.72) |
| Psychiatric disorders | Irritability | 19 | 6.34(4.04,9.96) | 2.66(0.99) |
| Psychiatric disorders | Schizophrenia | 19 | 18.21(11.59,28.6) | 4.17(2.51) |
| Nervous system disorders | Neuroleptic malignant syndrome^c^ | 19 | 30.69(19.53,48.25) | 4.92(3.26) |
| Nervous system disorders | Dyskinesia^c^ | 18 | 6.81(4.28,10.82) | 2.76(1.09) |
| Psychiatric disorders | Restlessness | 18 | 7.8(4.91,12.4) | 2.96(1.29) |
| Nervous system disorders | Extrapyramidal disorder^a^ | 17 | 26.36(16.35,42.5) | 4.71(3.04) |
| Nervous system disorders | Dysarthria | 17 | 7.59(4.71,12.23) | 2.92(1.25) |
| Nervous system disorders | Dystonia^a^ | 16 | 18.22(11.14,29.8) | 4.18(2.51) |
| Psychiatric disorders | Anger | 16 | 8.65(5.29,14.13) | 3.1(1.44) |
| General disorders and administration site conditions | Feeling drunk | 16 | 42.84(26.15,70.17) | 5.4(3.73) |
| Ear and labyrinth disorders | Vertigo | 15 | 3.89(2.34,6.46) | 1.96(0.29) |
| General disorders and administration site conditions | Influenza like illness | 15 | 3.46(2.09,5.75) | 1.79(0.12) |
| Musculoskeletal and connective tissue disorders | Muscle twitching | 14 | 9.73(5.75,16.45) | 3.27(1.61) |
| Vascular disorders | Orthostatic hypotension^c^ | 12 | 9.25(5.25,16.31) | 3.2(1.54) |
| Psychiatric disorders | Middle insomnia | 12 | 10.01(5.68,17.65) | 3.32(1.65) |
| Nervous system disorders | Cognitive disorder^c^ | 12 | 3.38(1.92,5.96) | 1.75(0.09) |
| General disorders and administration site conditions | Temperature regulation disorder | 12 | 61.82(34.93,109.42) | 5.92(4.25) |
| Nervous system disorders | Lethargy | 11 | 3.23(1.79,5.84) | 1.69(0.02) |
| Psychiatric disorders | Thinking abnormal | 11 | 10.27(5.68,18.56) | 3.35(1.69) |
| Nervous system disorders | Serotonin syndrome | 11 | 8.69(4.81,15.72) | 3.11(1.45) |
| General disorders and administration site conditions | Hangover | 11 | 52.29(28.83,94.87) | 5.69(4.02) |
| Psychiatric disorders | Delusion | 10 | 9.53(5.12,17.74) | 3.25(1.58) |
| Psychiatric disorders | Hallucination, visual | 10 | 6.44(3.46,11.99) | 2.68(1.02) |
| Musculoskeletal and connective tissue disorders | Muscle tightness | 10 | 10(5.37,18.61) | 3.32(1.65) |
| Psychiatric disorders | Fear | 10 | 8.07(4.33,15.01) | 3.01(1.34) |
| Psychiatric disorders | Disorientation | 10 | 4.63(2.49,8.61) | 2.21(0.54) |
| Eye disorders | Eye movement disorder | 9 | 22.54(11.7,43.43) | 4.48(2.82) |
| Psychiatric disorders | Euphoric mood | 9 | 14.96(7.77,28.81) | 3.9(2.23) |
| Nervous system disorders | Dysstasia | 9 | 4.16(2.16,8.01) | 2.05(0.39) |
| Psychiatric disorders | Emotional disorder | 8 | 4.18(2.09,8.37) | 2.06(0.39) |
| General disorders and administration site conditions | Crying | 8 | 4.25(2.12,8.51) | 2.08(0.42) |
| Nervous system disorders | Restless legs syndrome | 8 | 6.39(3.19,12.78) | 2.67(1) |
| Renal and urinary disorders | Urinary incontinence | 8 | 4.29(2.14,8.59) | 2.1(0.43) |
| Psychiatric disorders | Catatonia | 8 | 23.86(11.9,47.84) | 4.57(2.9) |
| Gastrointestinal disorders | Swollen tongue | 8 | 4.53(2.26,9.07) | 2.18(0.51) |
| Renal and urinary disorders | Urinary retention | 8 | 3.59(1.8,7.19) | 1.84(0.18) |
| Psychiatric disorders | Abnormal behaviour | 7 | 4.43(2.11,9.3) | 2.14(0.48) |
| Psychiatric disorders | Mood swings | 7 | 4.23(2.02,8.89) | 2.08(0.41) |
| Psychiatric disorders | Nightmare | 7 | 3.76(1.79,7.89) | 1.91(0.24) |
| Nervous system disorders | Dizziness postural | 7 | 9.31(4.43,19.56) | 3.21(1.55) |
| Eye disorders | Diplopia | 7 | 4.36(2.08,9.15) | 2.12(0.45) |
| Nervous system disorders | Movement disorder^c^ | 7 | 3.28(1.56,6.89) | 1.71(0.04) |
| Vascular disorders | Peripheral coldness | 7 | 7.46(3.55,15.67) | 2.9(1.23) |
| Reproductive system and breast disorders | Sexual dysfunction | 6 | 8.13(3.65,18.13) | 3.02(1.35) |
| Respiratory, thoracic and mediastinal disorders | Throat tightness | 6 | 3.49(1.57,7.77) | 1.8(0.13) |
| Psychiatric disorders | Abnormal dreams | 6 | 5.58(2.5,12.43) | 2.48(0.81) |
| Psychiatric disorders | Hypomania | 6 | 34.74(15.54,77.64) | 5.1(3.43) |
| Psychiatric disorders | Apathy | 5 | 5.79(2.41,13.93) | 2.53(0.86) |
| Gastrointestinal disorders | Tongue movement disturbance | 5 | 60.5(25,146.41) | 5.9(4.22) |
| Psychiatric disorders | Panic reaction | 5 | 10.76(4.47,25.89) | 3.42(1.75) |
| Psychiatric disorders | Self-injurious ideation | 5 | 20.93(8.69,50.42) | 4.38(2.71) |
| Psychiatric disorders | Dysphemia | 5 | 18.27(7.58,44) | 4.18(2.51) |
| Psychiatric disorders | Bruxism | 5 | 15.44(6.41,37.17) | 3.94(2.27) |
| Psychiatric disorders | Bipolar disorder | 5 | 7.89(3.28,18.99) | 2.98(1.31) |
| Nervous system disorders | Coordination abnormal | 5 | 8.34(3.47,20.06) | 3.06(1.39) |
| General disorders and administration site conditions | Thirst | 5 | 3.6(1.5,8.65) | 1.84(0.18) |
| Nervous system disorders | Sensory disturbance | 5 | 5.82(2.42,13.99) | 2.54(0.87) |
| Nervous system disorders | Electric shock sensation | 5 | 14.95(6.21,35.99) | 3.9(2.23) |
| Nervous system disorders | Drooling | 5 | 10.81(4.49,26.02) | 3.43(1.76) |
| Psychiatric disorders | initial insomnia | 5 | 8.29(3.44,19.94) | 3.05(1.38) |
| Investigations | Body temperature increased^c^ | 5 | 3.2(1.33,7.7) | 1.68(0.01) |
| Injury, poisoning and procedural complications | Sedation complication | 5 | 14.26(5.92,34.33) | 3.83(2.16) |
| Musculoskeletal and connective tissue disorders | Muscle rigidity | 4 | 4.74(1.78,12.65) | 2.24(0.58) |
| Psychiatric disorders | Soliloquy | 4 | 65.82(24.49,176.89) | 6.02(4.34) |
| Psychiatric disorders | Bradyphrenia | 4 | 7.72(2.89,20.59) | 2.94(1.28) |
| Psychiatric disorders | Somnambulism | 4 | 12.49(4.68,33.33) | 3.64(1.97) |
| Renal and urinary disorders | Incontinence | 4 | 5.79(2.17,15.44) | 2.53(0.86) |
| Nervous system disorders | Formication | 4 | 11.15(4.18,29.76) | 3.47(1.81) |
| General disorders and administration site conditions | Feeling of body temperature change | 4 | 10.54(3.95,28.12) | 3.39(1.72) |
| Psychiatric disorders | Personality change | 3 | 5.33(1.72,16.53) | 2.41(0.74) |
| Social circumstances | Refusal of treatment by patient | 3 | 10.84(3.49,33.69) | 3.43(1.77) |
| Musculoskeletal and connective tissue disorders | Posture abnormal | 3 | 14.26(4.59,44.31) | 3.83(2.16) |
| General disorders and administration site conditions | Screaming | 3 | 9.98(3.21,31) | 3.31(1.65) |
| Psychiatric disorders | Schizoaffective disorder bipolar type | 3 | 135.43(42.82,428.37) | 7.03(5.34) |
| Psychiatric disorders | Enuresis | 3 | 11.99(3.86,37.27) | 3.58(1.91) |
| Nervous system disorders | Slow speech | 3 | 16.69(5.37,51.88) | 4.05(2.38) |
| Respiratory, thoracic and mediastinal disorders | Hiccups | 3 | 5.86(1.89,18.18) | 2.55(0.88) |
| Psychiatric disorders | Trance | 3 | 176.47(55.47,561.47) | 7.4(5.7) |
| Nervous system disorders | Paralysis | 3 | 3.23(1.04,10.03) | 1.69(0.02) |
| Psychiatric disorders | Logorrhoea | 3 | 19(6.11,59.1) | 4.24(2.57) |
| Gastrointestinal disorders | Vomiting projectile | 3 | 17.54(5.64,54.54) | 4.13(2.46) |
| Psychiatric disorders | Tic | 3 | 10.07(3.24,31.3) | 3.33(1.66) |
| Product issues | Product packaging difficult to open | 3 | 8.84(2.85,27.44) | 3.14(1.47) |
| Nervous system disorders | Sleep paralysis | 3 | 31.06(9.97,96.77) | 4.94(3.27) |
| Skin and subcutaneous tissue disorders | Skin warm | 3 | 7.99(2.57,24.82) | 3(1.33) |
| Psychiatric disorders | Intrusive thoughts | 3 | 27.02(8.68,84.15) | 4.75(3.07) |
| Nervous system disorders | Pseudostroke | 3 | 122.6(38.83,387.08) | 6.89(5.2) |
| Investigations | Blood triglycerides increased | 3 | 3.88(1.25,12.03) | 1.95(0.29) |
| Musculoskeletal and connective tissue disorders | Muscle fatigue | 3 | 10.6(3.41,32.92) | 3.4(1.73) |
| Psychiatric disorders | Tachyphrenia | 3 | 15.7(5.05,48.8) | 3.97(2.3) |
| Psychiatric disorders | Anorgasmia | 3 | 20.72(6.66,64.47) | 4.37(2.69) |
| Psychiatric disorders | Autoscopy | 3 | 48.73(15.6,152.23) | 5.59(3.91) |
| Investigations | Drug screen positive | 3 | 14.31(4.6,44.47) | 3.83(2.16) |
| Psychiatric disorders | Psychotic symptom | 3 | 14.33(4.61,44.53) | 3.83(2.17) |
| Psychiatric disorders | Alcoholism | 3 | 7.44(2.4,23.09) | 2.89(1.22) |
| Psychiatric disorders | Homicidal ideation | 3 | 24.37(7.83,75.85) | 4.6(2.93) |

Abbreviation: n, Number of cases reporting PT; ROR, Reporting odds ratio; CI, confdence interval; IC, information component; IC 025, the lower 95% CI of IC

^a^, The PTs listed were existing in the Clinical Trials Experience

^b^,The PTs listed were existing in the Postmarketing Experience

^c^, The PTs listed were existing in the warning section of the drug label

Supplementary table 4a. Safety signals of lumateperone in females.

| SOC Name | Preferred terms (PTs) | n | ROR  (95% Two Sided CI) | IC(IC025) |
| --- | --- | --- | --- | --- |
| Nervous system disorders | Dizziness | 143 | 6.87(5.8,8.12) | 2.71(1.05) |
| General disorders and administration site conditions | Feeling abnormal | 70 | 6.19(4.89,7.85) | 2.6(0.93) |
| Nervous system disorders | Somnolence | 68 | 8.7(6.84,11.07) | 3.09(1.42) |
| Nervous system disorders | Burning sensation | 46 | 14.27(10.66,19.11) | 3.81(2.14) |
| Nervous system disorders | Sedation | 40 | 42.01(30.69,57.5) | 5.36(3.69) |
| Psychiatric disorders | Insomnia | 36 | 3.37(2.42,4.68) | 1.74(0.07) |
| Nervous system disorders | Tardive dyskinesia | 30 | 77.55(53.91,111.56) | 6.23(4.56) |
| Gastrointestinal disorders | Dry mouth | 30 | 8.67(6.05,12.43) | 3.1(1.43) |
| Psychiatric disorders | Mania | 26 | 56.3(38.15,83.08) | 5.78(4.11) |
| Psychiatric disorders | Suicidal ideation | 26 | 8.57(5.82,12.62) | 3.08(1.42) |
| general disorders and administration site conditions | Feeling hot | 25 | 7.94(5.35,11.78) | 2.98(1.31) |
| general disorders and administration site conditions | Unevaluable event | 25 | 7.91(5.34,11.74) | 2.97(1.3) |
| Nervous system disorders | Migraine | 25 | 4.05(2.73,6.01) | 2.01(0.34) |
| Nervous system disorders | Paraesthesia | 25 | 3.27(2.21,4.85) | 1.7(0.03) |
| General disorders and administration site conditions | Performance status decreased | 24 | 214.29(141.7,324.07) | 7.65(5.97) |
| Skin and subcutaneous tissue disorders | Skin burning sensation | 19 | 4.11(2.62,6.45) | 2.03(0.36) |
| Psychiatric disorders | Psychotic disorder | 18 | 23.63(14.84,37.62) | 4.54(2.88) |
| Psychiatric disorders | Hallucination | 18 | 6.72(4.22,10.68) | 2.74(1.07) |
| Nervous system disorders | Balance disorder | 18 | 4.78(3,7.6) | 2.25(0.58) |
| General disorders and administration site conditions | Chills | 18 | 3.42(2.15,5.43) | 1.77(0.1) |
| Psychiatric disorders | Hallucination, auditory | 16 | 30(18.31,49.14) | 4.89(3.22) |
| Nervous system disorders | Hypersomnia | 15 | 12.44(7.48,20.69) | 3.63(1.96) |
| Nervous system disorders | Loss of consciousness | 15 | 3.24(1.95,5.39) | 1.69(0.02) |
| Psychiatric disorders | Agitation | 14 | 7.09(4.19,11.99) | 2.82(1.15) |
| Gastrointestinal disorders | Dysphagia | 14 | 4.16(2.46,7.04) | 2.05(0.38) |
| General disorders and administration site conditions | Feeling drunk | 13 | 54.45(31.45,94.28) | 5.74(4.07) |
| General disorders and administration site conditions | Feeling cold | 13 | 9.96(5.77,17.19) | 3.31(1.64) |
| Psychiatric disorders | Irritability | 13 | 7.94(4.6,13.7) | 2.98(1.31) |
| Nervous system disorders | Akathisia | 12 | 28.25(15.99,49.92) | 4.8(3.14) |
| Nervous system disorders | Dysarthria | 12 | 9.64(5.47,17.01) | 3.26(1.59) |
| Psychiatric disorders | Restlessness | 12 | 9.24(5.24,16.3) | 3.2(1.53) |
| Nervous system disorders | Disturbance in attention | 12 | 6.01(3.41,10.59) | 2.58(0.91) |
| Nervous system disorders | Amnesia | 12 | 4.9(2.78,8.64) | 2.29(0.62) |
| Nervous system disorders | Neuroleptic malignant syndrome | 11 | 47.33(26.08,85.9) | 5.54(3.87) |
| Psychiatric disorders | Paranoia | 11 | 23.17(12.79,41.97) | 4.52(2.85) |
| Nervous system disorders | Dystonia | 10 | 19.84(10.64,36.97) | 4.3(2.63) |
| Psychiatric disorders | Middle insomnia | 10 | 12.76(6.85,23.77) | 3.66(2) |
| Psychiatric disorders | Disorientation | 10 | 8.33(4.47,15.5) | 3.05(1.38) |
| General disorders and administration site conditions | Temperature regulation disorder | 9 | 62.47(32.28,120.88) | 5.94(4.26) |
| Nervous system disorders | Extrapyramidal disorder | 9 | 29.55(15.32,57) | 4.87(3.2) |
| Nervous system disorders | Serotonin syndrome | 8 | 10.59(5.29,21.23) | 3.4(1.73) |
| Psychiatric disorders | Anger | 8 | 8.66(4.32,17.35) | 3.11(1.44) |
| Nervous system disorders | Dysstasia | 8 | 5.57(2.78,11.15) | 2.47(0.8) |
| Nervous system disorders | Cognitive disorder | 8 | 3.69(1.84,7.39) | 1.88(0.21) |
| Vascular disorders | Orthostatic hypotension | 7 | 10.76(5.12,22.62) | 3.42(1.75) |
| Musculoskeletal and connective tissue disorders | Muscle tightness | 7 | 10.13(4.82,21.29) | 3.33(1.67) |
| Psychiatric disorders | Aggression | 7 | 7.55(3.59,15.86) | 2.91(1.24) |
| Renal and urinary disorders | Urinary incontinence | 7 | 5.72(2.72,12.01) | 2.51(0.84) |
| Gastrointestinal disorders | Swollen tongue | 7 | 5.37(2.56,11.29) | 2.42(0.75) |
| Nervous system disorders | Dyskinesia | 7 | 4.72(2.25,9.92) | 2.24(0.57) |
| Eye disorders | Eye movement disorder | 6 | 25.4(11.37,56.75) | 4.65(2.98) |
| Psychiatric disorders | Schizophrenia | 6 | 12.87(5.77,28.71) | 3.68(2.01) |
| Psychiatric disorders | Thinking abnormal | 6 | 8.81(3.95,19.65) | 3.13(1.47) |
| Psychiatric disorders | Hallucination, visual | 6 | 7.99(3.58,17.82) | 2.99(1.33) |
| Nervous system disorders | Restless legs syndrome | 6 | 7.46(3.35,16.64) | 2.89(1.23) |
| Injury, poisoning and procedural complications | Head injury | 6 | 4.86(2.18,10.83) | 2.28(0.61) |
| General disorders and administration site conditions | Hangover | 5 | 39.39(16.3,95.16) | 5.28(3.61) |
| Psychiatric disorders | Catatonia | 5 | 32.66(13.53,78.85) | 5.01(3.34) |
| Nervous system disorders | Dizziness postural | 5 | 12.68(5.27,30.54) | 3.66(1.99) |
| Psychiatric disorders | Abnormal dreams | 5 | 8.49(3.53,20.43) | 3.08(1.41) |
| Vascular disorders | Peripheral coldness | 5 | 8.45(3.51,20.35) | 3.07(1.41) |
| Psychiatric disorders | Fear | 5 | 5.81(2.42,13.99) | 2.54(0.87) |
| Musculoskeletal and connective tissue disorders | Muscle twitching | 5 | 5.48(2.28,13.19) | 2.45(0.78) |
| Psychiatric disorders | Emotional disorder | 5 | 5.18(2.15,12.45) | 2.37(0.7) |
| Psychiatric disorders | Mood swings | 5 | 4.64(1.93,11.17) | 2.21(0.54) |
| Nervous system disorders | Movement disorder | 5 | 3.55(1.48,8.54) | 1.83(0.16) |
| General disorders and administration site conditions | Crying | 5 | 3.42(1.42,8.24) | 1.77(0.11) |
| Psychiatric disorders | Hypomania | 4 | 34.68(12.94,92.91) | 5.1(3.43) |
| Nervous system disorders | Electric shock sensation | 4 | 15.82(5.92,42.27) | 3.98(2.31) |
| Nervous system disorders | Formication | 4 | 14.6(5.47,39.02) | 3.86(2.19) |
| General disorders and administration site conditions | Feeling of body temperature change | 4 | 13.94(5.22,37.24) | 3.79(2.12) |
| Nervous system disorders | Sensory disturbance | 4 | 6.51(2.44,17.36) | 2.7(1.03) |
| Nervous system disorders | Mental impairment | 4 | 4.33(1.62,11.57) | 2.11(0.45) |
| Eye disorders | Diplopia | 4 | 4.18(1.57,11.16) | 2.06(0.39) |
| Investigations | Body temperature increased | 4 | 3.98(1.49,10.61) | 1.99(0.32) |
| Psychiatric disorders | Trance | 3 | 340.53(104.05,1114.5) | 8.28(6.54) |
| Nervous system disorders | Pseudostroke | 3 | 251.34(77.86,811.36) | 7.87(6.15) |
| Psychiatric disorders | Autoscopy | 3 | 83.78(26.65,263.43) | 6.35(4.67) |
| Gastrointestinal disorders | Tongue movement disturbance | 3 | 62.83(20.05,196.91) | 5.95(4.27) |
| Psychiatric disorders | Anorgasmia | 3 | 44.54(14.25,139.18) | 5.46(3.78) |
| Psychiatric disorders | Intrusive thoughts | 3 | 44.35(14.19,138.59) | 5.45(3.77) |
| Nervous system disorders | Sleep paralysis | 3 | 43.44(13.9,135.72) | 5.42(3.75) |
| Psychiatric disorders | Tachyphrenia | 3 | 28.61(9.18,89.16) | 4.83(3.15) |
| Gastrointestinal disorders | Vomiting projectile | 3 | 23.88(7.67,74.38) | 4.57(2.89) |
| Psychiatric disorders | Enuresis | 3 | 23.35(7.5,72.73) | 4.54(2.86) |
| Psychiatric disorders | Self-injurious ideation | 3 | 19.44(6.25,60.5) | 4.27(2.6) |
| Psychiatric disorders | Dysphemia | 3 | 17.74(5.7,55.2) | 4.14(2.47) |
| Injury, poisoning and procedural complications | Sedation complication | 3 | 13.1(4.21,40.72) | 3.7(2.03) |
| Psychiatric disorders | Euphoric mood | 3 | 9.72(3.13,30.2) | 3.28(1.61) |
| Psychiatric disorders | Panic reaction | 3 | 8.44(2.72,26.21) | 3.07(1.4) |
| Nervous system disorders | Coordination abnormal | 3 | 8.04(2.59,24.97) | 3(1.33) |
| Psychiatric disorders | Apathy | 3 | 6.39(2.06,19.83) | 2.67(1) |
| Psychiatric disorders | Delusion | 3 | 6.14(1.98,19.07) | 2.61(0.95) |
| Metabolism and nutrition disorders | Increased appetite | 3 | 4.74(1.53,14.71) | 2.24(0.57) |
| Gastrointestinal disorders | Paraesthesia oral | 3 | 3.88(1.25,12.05) | 1.95(0.29) |
| Social circumstances | Impaired work ability | 3 | 3.63(1.17,11.27) | 1.86(0.19) |

Abbreviation: n, Number of cases reporting PT; ROR, Reporting odds ratio; CI, confdence interval; IC, information component; IC 025, the lower 95% CI of IC

Supplementary table 4b. Safety signals of lumateperone in males.

| SOC Name | Preferred terms (PTs) | n | ROR  (95% Two Sided CI) | IC(IC025) |
| --- | --- | --- | --- | --- |
| Nervous system disorders | Dizziness | 42 | 4.36(3.21,5.93) | 2.09(0.43) |
| Nervous system disorders | Somnolence | 41 | 9.74(7.14,13.29) | 3.25(1.58) |
| Gastrointestinal disorders | Nausea | 40 | 3.26(2.38,4.47) | 1.68(0.01) |
| Nervous system disorders | Headache | 35 | 3.57(2.55,4.99) | 1.81(0.14) |
| Psychiatric disorders | Insomnia | 31 | 6.18(4.33,8.82) | 2.6(0.93) |
| Psychiatric disorders | Suicidal ideation | 26 | 14.02(9.51,20.67) | 3.78(2.11) |
| Psychiatric disorders | Mania | 25 | 84.65(56.79,126.19) | 6.35(4.68) |
| Nervous system disorders | Sedation | 25 | 40.53(27.24,60.28) | 5.3(3.64) |
| General disorders and administration site conditions | Feeling abnormal | 25 | 5.73(3.86,8.51) | 2.5(0.83) |
| Gastrointestinal disorders | Dry mouth | 21 | 16.23(10.54,24.98) | 4(2.33) |
| General disorders and administration site conditions | Unevaluable event | 21 | 11.16(7.25,17.17) | 3.46(1.79) |
| Psychiatric disorders | Anxiety | 21 | 3.21(2.09,4.94) | 1.67(0) |
| Nervous system disorders | Tardive dyskinesia | 20 | 84.44(54.07,131.85) | 6.35(4.68) |
| Psychiatric disorders | Hallucination, auditory | 20 | 49.73(31.91,77.52) | 5.6(3.93) |
| Psychiatric disorders | Psychotic disorder | 19 | 26.03(16.53,40.98) | 4.68(3.01) |
| Psychiatric disorders | Aggression | 18 | 13.32(8.36,21.21) | 3.72(2.05) |
| Nervous system disorders | Burning sensation | 17 | 16.5(10.22,26.64) | 4.02(2.36) |
| General disorders and administration site conditions | Performance status decreased | 14 | 149.82(87.67,256.03) | 7.16(5.49) |
| Nervous system disorders | Tremor | 14 | 4.37(2.58,7.39) | 2.12(0.45) |
| Psychiatric disorders | Schizophrenia | 13 | 24.6(14.23,42.54) | 4.6(2.93) |
| Psychiatric disorders | Paranoia | 12 | 29.89(16.9,52.86) | 4.88(3.21) |
| Nervous system disorders | Hypersomnia | 11 | 16.15(8.91,29.25) | 4(2.33) |
| Psychiatric disorders | Agitation | 11 | 6.7(3.7,12.12) | 2.73(1.06) |
| Nervous system disorders | Dyskinesia | 10 | 10.42(5.59,19.43) | 3.37(1.7) |
| Skin and subcutaneous tissue disorders | Skin burning sensation | 10 | 9.7(5.2,18.07) | 3.27(1.6) |
| Nervous system disorders | Akathisia | 8 | 25.88(12.89,51.96) | 4.68(3.01) |
| Musculoskeletal and connective tissue disorders | Muscle twitching | 8 | 17.4(8.67,34.9) | 4.11(2.44) |
| Nervous system disorders | Disturbance in attention | 8 | 6.56(3.27,13.15) | 2.71(1.04) |
| Psychiatric disorders | Suicide attempt | 8 | 5.99(2.99,12.01) | 2.58(0.91) |
| Nervous system disorders | Syncope | 8 | 3.53(1.76,7.07) | 1.81(0.14) |
| Psychiatric disorders | Delusion | 7 | 13.3(6.32,27.98) | 3.72(2.05) |
| Psychiatric disorders | Anger | 7 | 8.77(4.17,18.44) | 3.12(1.46) |
| Nervous system disorders | Extrapyramidal disorder | 6 | 21.58(9.66,48.21) | 4.42(2.75) |
| Nervous system disorders | Neuroleptic malignant syndrome | 6 | 17.18(7.69,38.36) | 4.09(2.42) |
| Reproductive system and breast disorders | Sexual dysfunction | 6 | 13.69(6.13,30.56) | 3.77(2.1) |
| General disorders and administration site conditions | Feeling cold | 6 | 11.36(5.09,25.36) | 3.5(1.83) |
| Psychiatric disorders | Abnormal behaviour | 6 | 7.81(3.5,17.43) | 2.96(1.29) |
| General disorders and administration site conditions | Feeling hot | 6 | 5.82(2.61,12.98) | 2.53(0.87) |
| Vascular disorders | Flushing | 6 | 4.73(2.12,10.55) | 2.24(0.57) |
| Psychiatric disorders | Bipolar disorder | 5 | 23.65(9.8,57.05) | 4.55(2.88) |
| Nervous system disorders | Dystonia | 5 | 14.91(6.19,35.92) | 3.89(2.22) |
| Psychiatric disorders | Fear | 5 | 14.09(5.85,33.95) | 3.81(2.14) |
| Psychiatric disorders | Thinking abnormal | 5 | 14.04(5.83,33.83) | 3.8(2.13) |
| General disorders and administration site conditions | Therapeutic response unexpected | 5 | 6.51(2.7,15.67) | 2.7(1.03) |
| Nervous system disorders | Dysarthria | 5 | 5.63(2.34,13.56) | 2.49(0.82) |
| Psychiatric disorders | Restlessness | 5 | 5.47(2.27,13.18) | 2.45(0.78) |
| Renal and urinary disorders | Urinary retention | 5 | 4.64(1.93,11.16) | 2.21(0.54) |
| General disorders and administration site conditions | Influenza like illness | 5 | 4.46(1.85,10.75) | 2.15(0.49) |
| Psychiatric disorders | Stress | 5 | 3.73(1.55,8.97) | 1.89(0.23) |
| Psychiatric disorders | Hallucination, visual | 4 | 6.23(2.33,16.64) | 2.63(0.97) |
| Psychiatric disorders | Nightmare | 4 | 6.11(2.29,16.32) | 2.61(0.94) |
| Ear and labyrinth disorders | Vertigo | 4 | 4.18(1.57,11.17) | 2.06(0.39) |
| Nervous system disorders | Migraine | 4 | 3.92(1.47,10.47) | 1.97(0.3) |
| Psychiatric disorders | Irritability | 4 | 3.62(1.36,9.67) | 1.85(0.19) |
| General disorders and administration site conditions | Withdrawal syndrome | 4 | 3.26(1.22,8.7) | 1.7(0.03) |
| Nervous system disorders | Cognitive disorder | 4 | 3.19(1.19,8.51) | 1.67(0) |
| Psychiatric disorders | Schizoaffective disorder bipolar type | 3 | 304.91(94.13,987.71) | 8.14(6.42) |
| Psychiatric disorders | Homicidal ideation | 3 | 45.91(14.7,143.44) | 5.5(3.82) |
| General disorders and administration site conditions | Feeling drunk | 3 | 26.54(8.52,82.7) | 4.72(3.05) |
| Eye disorders | Eye movement disorder | 3 | 21.16(6.8,65.87) | 4.39(2.72) |
| Nervous system disorders | Drooling | 3 | 18.18(5.84,56.58) | 4.18(2.5) |
| Psychiatric disorders | Euphoric mood | 3 | 12.37(3.98,38.47) | 3.62(1.95) |
| Psychiatric disorders | Personality change | 3 | 12.19(3.92,37.92) | 3.6(1.93) |
| Respiratory, thoracic and mediastinal disorders | Throat tightness | 3 | 8.75(2.81,27.19) | 3.12(1.45) |
| Eye disorders | Diplopia | 3 | 5.81(1.87,18.05) | 2.53(0.87) |
| Vascular disorders | Orthostatic hypotension | 3 | 5.56(1.79,17.27) | 2.47(0.8) |
| Psychiatric disorders | Panic attack | 3 | 4.98(1.6,15.46) | 2.31(0.64) |
| Psychiatric disorders | Intentional self-injury | 3 | 4.86(1.57,15.11) | 2.28(0.61) |
| Metabolism and nutrition disorders | Hypophagia | 3 | 4.48(1.44,13.9) | 2.16(0.49) |
| Psychiatric disorders | Nervousness | 3 | 4.27(1.37,13.26) | 2.09(0.42) |
| Psychiatric disorders | Emotional disorder | 3 | 3.43(1.11,10.67) | 1.78(0.11) |

Abbreviation: n, Number of cases reporting PT; ROR, Reporting odds ratio; CI, confdence interval; IC, information component; IC 025, the lower 95% CI of IC
